# Isolated Cysticercosis Lump over Thigh in a Child

**Published:** 2015-05-01

**Authors:** Chandrashekhar A Sohoni

**Affiliations:** NM Medical, Radiology Department, Kalyani Nagar, Pune, Maharashtra, India

**Dear Sir,**

Cysticercosis is caused by the larval form of the parasite Taenia solium (T. solium). The central nervous system and orbits are the most frequently involved sites.[1-3] Isolated cysticercosis presenting as a lump in the lower extremity is very unusual in children and may present a diagnostic challenge.

A 6-year-old boy presented with a progressive lump along the anterolateral aspect of the left thigh for a month. There was no history of trauma, fever, anorexia, or weight loss. The patient was a non-vegetarian. The family of the patient was neither involved in pig-farming nor were wild boars common in the area of patient’s residence. On examination, the swelling was mildly tender, non-pulsatile, fluctuant, and transilluminant. Ultrasonography (USG) revealed a well-defined cystic lesion with internal echoes located in the superficial fibres of vastus lateralis muscle, measuring 4.0cm x 1.9cm x 1.5cm. The wall-thickness of the cyst measured 4mm and there was a tiny echogenic nodule along the anterior wall on the luminal side. MRI showed intermediate signal on T1W and hyperintense signal on T2W sequence, and also revealed sharp peripheral wall enhancement upon intravenous administration of gadolinium (Fig. 1). The tiny nodule along the luminal surface of the anterior wall appeared hypointense on all pulse-sequences and did not reveal enhancement on post-contrast images, suggestive of a scolex (Fig. 1). Based on the imaging findings, a diagnosis of isolated giant muscular cysticercosis was suggested. The Enzyme-linked Immunosorbent Assay (ELISA) titre for T. solium was raised (1:500). Haemogram was unremarkable, except for mild increase in differential eosinophil count (8%). ESR was mildly raised (20 mm/hr). Stool examination was normal.

**Figure:**
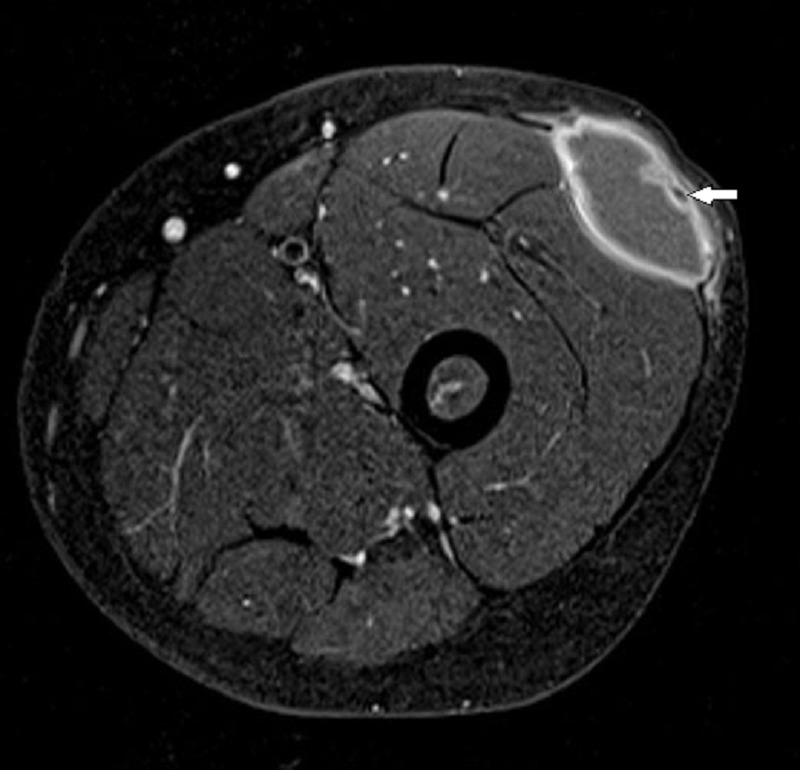
Figure 1: MRI showing lesion with post-contrast wall enhancement. Arrow showed scolex.

In view of the superficial location and relatively large size, surgical excision was advised. Since the parents did not agree for the same, albendazole 15 mg/kg/day in two divided doses was administered orally for 4 weeks and patient was closely monitored. A short, tapering course of prednisolone starting at 2mg/kg/day was also administered. At the end of 4 weeks, the swelling had completely subsided on palpation with no residual symptoms. An USG at this time showed complete regression of the cyst. Follow-up examination 2 months later did not reveal any evidence of recurrence.

Most cases of muscular cysticercosis are asymptomatic, but when it manifests clinically, it generally belongs to one of the three types – the myalgic type, the nodular type, or the pseudohypertrophy type.[4] Though histopathological analysis after fine needle aspiration cytology or biopsy provides the most conclusive evidence of muscular cysticercosis, imaging in combination with serology is usually sufficient to make a fairly accurate diagnosis.[5] Presence of a cyst with an eccentrically located scolex is the classical imaging appearance of muscular cysticercosis on USG as well as MRI. Nodular type cysticercosis, which was seen in our patient, is the result of degeneration of the cyst with intermittent leakage of fluid in the surrounding tissue resulting in perilesional inflammatory response. In addition to mild pain, the relatively thick wall around the cyst, presence of internal echoes on USG, and peripheral enhancement on post-contrast MRI were consistent with a degenerating cyst inciting an inflammatory response. USG alone can be suggestive of a diagnosis of muscular cysticercosis.[4,6] However, in addition to corroborating the USG diagnosis, MRI provides information regarding the exact location, extent and multiplicity of the lesions.[5] In our case, the relatively thick wall of the cyst and presence of internal echoes on ultrasound warranted further characterisation of the lesion with MRI, as cold abscess was another diagnostic consideration.[5,7]

Whenever feasible, surgical excision is advisable for symptomatic isolated muscular cysticercus lesions.[8] Where surgery is not possible, medical therapy with albendazole or praziquantel is recommended. A short course of steroids is also recommended to reduce the inflammatory reaction to antiparasitic treatment.[8]

## Footnotes

**Source of Support:** Nil

**Conflict of Interest:** None declared

